# Long-Term Survival of a Lynch Syndrome Patient With Eight Primary Tumors: A Case Report

**DOI:** 10.3389/fonc.2022.896024

**Published:** 2022-05-10

**Authors:** Jing Jiang, Ting Huang, Xianlei Lin, Yu Zhang, Xuefei Yang, Ling Huang, Zhifeng Ye, Xingchang Ren, Lisong Teng, Jun Li, Mei Kong, Liyan Lian, Jinhua Lu, Yazhen Zhong, Zechen Lin, Ming Xu, Yin Chen, Shengyou Lin

**Affiliations:** ^1^ Department of Oncology, Hangzhou Traditional Chinese Medicine (TCM) Hospital Affiliated to Zhejiang Chinese Medical University, Hangzhou, China; ^2^ The Third Clinical Medical College, Zhejiang Chinese Medical University, Hangzhou, China; ^3^ Department of Pathology, Hangzhou Traditional Chinese Medicine (TCM) hospital Affiliated to Zhejiang Chinese Medical University, Hangzhou, China; ^4^ Department of Surgical Oncology, The First Affiliated Hospital of Zhejiang University, Hangzhou, China; ^5^ Department of Pathology, The First Affiliated Hospital of Zhejiang University, Hangzhou, China

**Keywords:** multiple primary tumors, lynch syndrome, prostatic carcinoma, malignant peripheral nerve sheath tumor, long-term survival

## Abstract

With the modern technological developments in the diagnosis and treatment of cancer, the survival rate of cancer patients has increased. On the other hand, the incidence of multiple primary tumors is increasing annually. Lynch syndrome (LS), an autosomal dominant disorder with germline mutations in DNA mismatch repair genes, increases the risk of cancer in patients carrying those mutations. In this report, we present an extremely rare case of an 81-year-old male patient with eight primary malignancies and LS. The patient is still alive having survived for more than 41 years since the initial discovery of the first tumor. The eighth and most recently diagnosed primary cancer was a malignant peripheral nerve sheath tumor. Although there have been numerous reports of malignancies in LS, malignant peripheral nerve sheath tumors have not been reported previously with LS. Here, we report, to the best of our knowledge, the first case of a malignant peripheral nerve sheath tumor with LS.

## Introduction

Multiple primary tumors refer to the simultaneous or metachronous occurrence of two or more primary tumors in one or more tissues and organs in the same individual (1). With the development of cancer screening, diagnosis, and treatment modalities, the survival rate of patients with a first primary tumor has improved, but the incidence of multiple primary tumors is increasing. The incidence of multiple primary cancers is approximately 2–17% among cancer patients (2). A population-based study from the Netherlands reported that 6.5% of patients with multiple primary cancers had two primary cancers, 0.5% had three primary cancers, and 0.05% had four or more primary cancers (3). The current incidence of multiple primary tumors is still rare in clinical practice, especially the cases of three or more tumors.

Lynch syndrome (LS) is an autosomal dominant cancer-predisposing syndrome caused by germline mutations in the DNA mismatch repair (MMR) genes *MLH1, MSH2, MSH6, MSH2*, and *EPCAM*, and is characterized by microsatellite instability (MSI) in tumors. Carriers of pathogenic mutations in these genes, have an increased risk of colorectal cancer and other malignancies over their lifetime compared to the general population (4). LS is a high-risk factor for multiple primary tumors.

In this study, we present an extremely rare clinical case of a patient with LS who had eight primary tumors and survived for 41 years.

## Case Description

The patient is an 81-year-old male with multiple primary tumors (**Table 1**). The patient visited our hospital in December 2021 because of left colon carcinoma 40 years after surgery, carcinoma of the rectum 30 years after surgery, gastric carcinoma 23 years after surgery, transverse colonic carcinoma 12 years after surgery, nasal borderline tumor 11 years after surgery, skin carcinoma 10 years after surgery, diagnosis of prostatic carcinoma for 3 years, and a right inguinal mass for more than 3 months.

**Table 1:** Medical history.

Year	Age at diagnosis	Localization	Operation	Pathological type	MMR
1981	40	Left colon	Radical left hemicolectomy combined with splenectomy	Adenocarcinoma	No data
1991	50	Rectum	Mile’s radical resection	Adenocarcinoma	No data
1997	56	Stomach	radical gastrectomy	Adenocarcinoma	No data
2009	68	Transverse colon	radical resection of transverse colon cancer and ascending colostomy	Adenocarcinoma	No data
2010	69	Nasal cavity	nasal mucosal augmentation resection	Borderline tumor of the nasal mucosa	No data
2011	70	Abdominal skin	skin tumor resection	Squamous cell carcinoma	No data
2018	77	Prostate	None	Adenocarcinoma	MSH2/MSH6(-)
2022	81	right inguinal	None	spindle-cell tumors	MSH2/MSH6(-)

MMR, mismatch repair.

**Figure 1:**
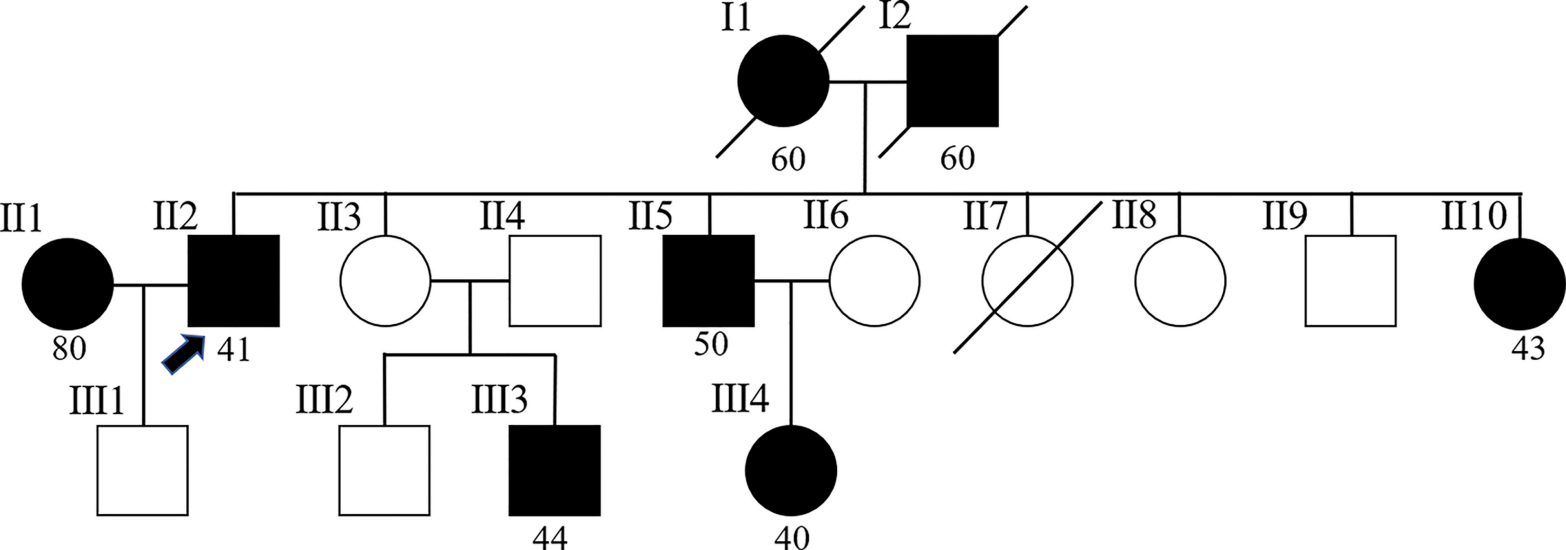
Pedigree of the family. The arrow (→) points to the proband. Squares and circles respectively represent males and females. Roman numerals indicate generations. Solid symbols represent cancer patients. Symbols with a slash indicate deceased individuals. The number below shows the initial onset age. Patient number II2 is the proband. Patients number I1and I2 suffered from colon cancer at the age of 60. Patient number II1 suffered from malignant melanoma of the perineum at the age of 80. Patient number II5 suffered from colon cancer at the age of 50. Patients number II10 and III3 suffered from colon cancer before the age of 50. Furthermore, patient number II10 also suffered from transverse colon carcinoma at the age of 57. Patient number III4 suffered from ovarian cancer at 40, and breast carcinoma at 47.

The patient was a retired professional technician living in Hangzhou, Zhejiang Province, and had good living conditions. The patient had no history of smoking or drinking, and no history of excessive sun exposure. He was married at 27 years old and has a son in good health. He has a harmonious family relationship and has no bad psychological problems since his illness. His wife was diagnosed with malignant melanoma of the perineum in December 2021. In terms of the patient’s family history, his father suffered from colon cancer at the age of 60, his mother was diagnosed with colon cancer in the same year, and both of them passed away. One younger brother and one younger sister suffered from colon cancer in their 50s. Furthermore, this younger sister also suffered from transverse colon carcinoma at the age of 57. In addition, a son of one of his sisters was also diagnosed with colon cancer, and a daughter of one of his brothers was also diagnosed with ovarian cancer at 40, and breast carcinoma at 47 (**Figure 1**).

In 1981, our patient presented to the First Affiliated Hospital of Zhejiang University with an abdominal discomfort. Gastrointestinal endoscopy revealed left semicolon cancer. The patient underwent radical left hemicolectomy combined with splenectomy, and the postoperative pathological diagnosis was moderately differentiated adenocarcinoma of the colon. In 1991, he was diagnosed with a rectal mass on gastrointestinal endoscopy. The patient underwent Mile’s radical resection for rectal cancer, and postoperative pathology revealed poorly differentiated adenocarcinoma of rectum. No further treatment was administered. Since then, he has undergone annual regular gastrointestinal endoscopy. In 1997, a gastric space-occupying mass was found during a regular gastrointestinal endoscopy. The patient underwent radical gastrectomy for gastric cancer, and postoperative pathology revealed gastric ulcer type poorly differentiated adenocarcinoma. In 2009, the patient underwent radical resection of transverse colon cancer and ascending colostomy. Postoperative pathology showed poorly differentiated adenocarcinoma of transverse colon. One year later, due to poor nasal ventilation, the patient took nasal endoscopy. Nasal endoscopy revealed the right nasal vestibule vegetation. He underwent nasal mucosal augmentation resection. Postoperative pathology revealed a borderline tumor of the nasal mucosa. In 2011, a skin biopsy was performed as rough protrusion of the abdominal skin with pigment changes were observed. He was diagnosed with squamous cell carcinoma *in situ*, and surgical resection was performed.

**Figure 2:**
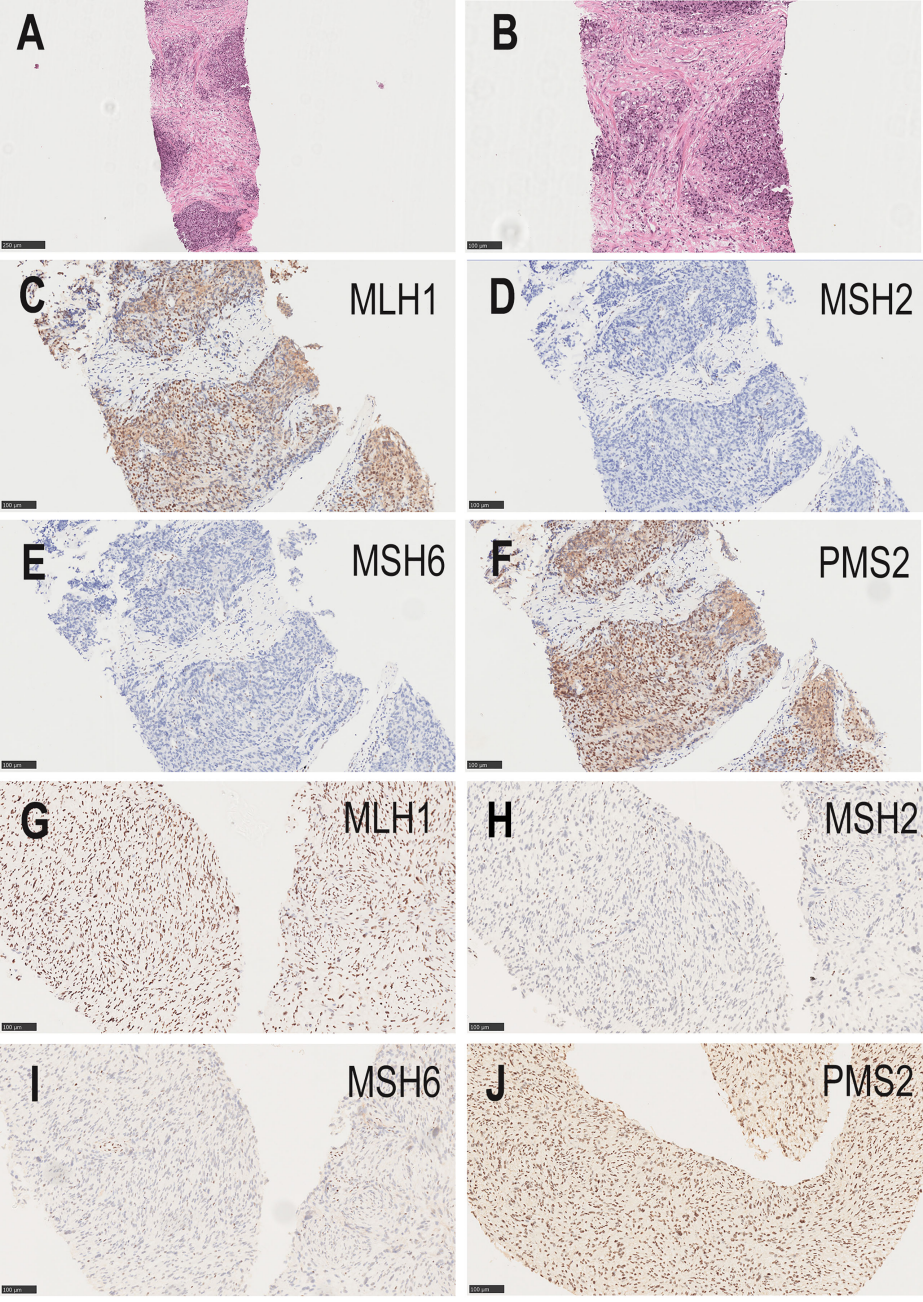
**(A, B)** The needle core biopsies of the prostate show a high-grade prostatic adenocarcinoma. (**A**, hematoxylin–eosin×100; **B**, hematoxylin–eosin×200). **(C–F)** Absence of MSH2 and MSH6 staining, and positive staining for MLH1and PMS2 in the prostatic tumor (original magnifications ×200). **(G–J)** Absence of MSH2 and MSH6 staining, and positive staining for MLH1and PMS2 in the malignant peripheral nerve sheath tumor (original magnifications ×200).

In February 2018, the patient presented to our hospital with complaints of poor urination. On routine ultrasonography, the urologist detected a lump in the patient’s prostate. The total serum prostate-specific antigen (PSA) level was 40 ng/mL, and needle core biopsy was performed. Histopathology revealed high-grade prostatic adenocarcinoma (**Figures 2A, B**), and Gleason Score 4 + 5 = 9. The tumor was immunohistochemically positive for P504S and negative for CK (34Be12), P63, and Syn. In terms of treatment, he first received endocrine therapy (goserelin acetate sustained-release depot combined with bicalutamide tablets) and then external radiotherapy was applied. At the initial stage of treatment, there was a significant reduction in the tumor focus, and urination became unobstructed. In September of the same year, the patient’s serum PSA level increased again. Considering the resistance to first-line endocrine therapy, the patient was given second-line endocrine therapy (abiraterone acetate combined with prednisone). After 5 months of treatment, the serum PSA level increased again. The patient was considered to be resistant to endocrine therapy for prostate cancer. Consequently, the patient underwent four rounds of chemotherapy (docetaxel combined with prednisone); however, the disease progressed again. The patient had significant edema of the left lower extremity caused by tumor compression in the pelvic cavity; thus, pelvic radiotherapy was repeated. Using targeted next-generation sequencing (NGS) technology, quantitative paired plasma and leukocyte samples from the patient were sequenced with a specific 425-gene panel. The 425 cancer-related genes included genes with Food and Drug Administration(FDA)-approved targeted medicine and Chemotherapeutic medicine, and National Comprehensive Cancer Network(NCCN) guideline recommendations. Genetic risk-related mutated genes were also included in the test. The captured samples were then subjected to the Illumina HiSeq 4000 platform (Illumina, San Diego, CA) for sequencing, with a mean depth of 500× for FFPE samples and 3,000× for ctDNA samples from CSF or plasma. Copy number variations (CNVs) were detected using CNV Kit. CNV gain and loss were identified if depth ratio were above 1.6 or below 0.6, respectively. And gene feature information was obtained from the Clinical Testing Center (Nanjing Geneseeq Technology Inc., China). NGS revealed that the patient had 128 tumor-specific mutations in peripheral blood genes, including mutations in the MMR genes *MLH1* and *MLH3*, in addition to mutations in *BRCA1* and *TP53*, and a germline deletion in exon 12 of *MSH2*, with a high tumor mutational burden of 139.1 mutations/MB. Based on the NGS results of a germline deletion in *MSH2* combined with the patient’s previous cancer history and family cancer history, the patient was diagnosed with LS. Genetic counseling was recommended for his relatives at the same time.

To further define the relationship between prostate cancer and LS, we performed MMR protein-related immunohistochemistry. Immunohistochemistry for prostate cancer was positive for MLH1 and PMS2, but negative for MSH2 and MSH6 (**Figures 2C**–**F**). These results confirmed LS-associated prostate cancer. According to the clinical treatment guidelines, the patient was given intravenous infusions of the PD-1 inhibitor, pembrolizumab (200 mg every 3 weeks) (Merck & Co., Inc.), combined with oral anlotinib hydrochloride (8 mg once daily, Days 1–14, 1 cycle every 3 weeks) (Jiangsu Chiatai Tianqing Medicine Co., Ltd). The serum PSA level was within the normal range during treatment, and computed tomography (CT) showed no significant enlargement of the prostate tumor.

**Figure 3:**
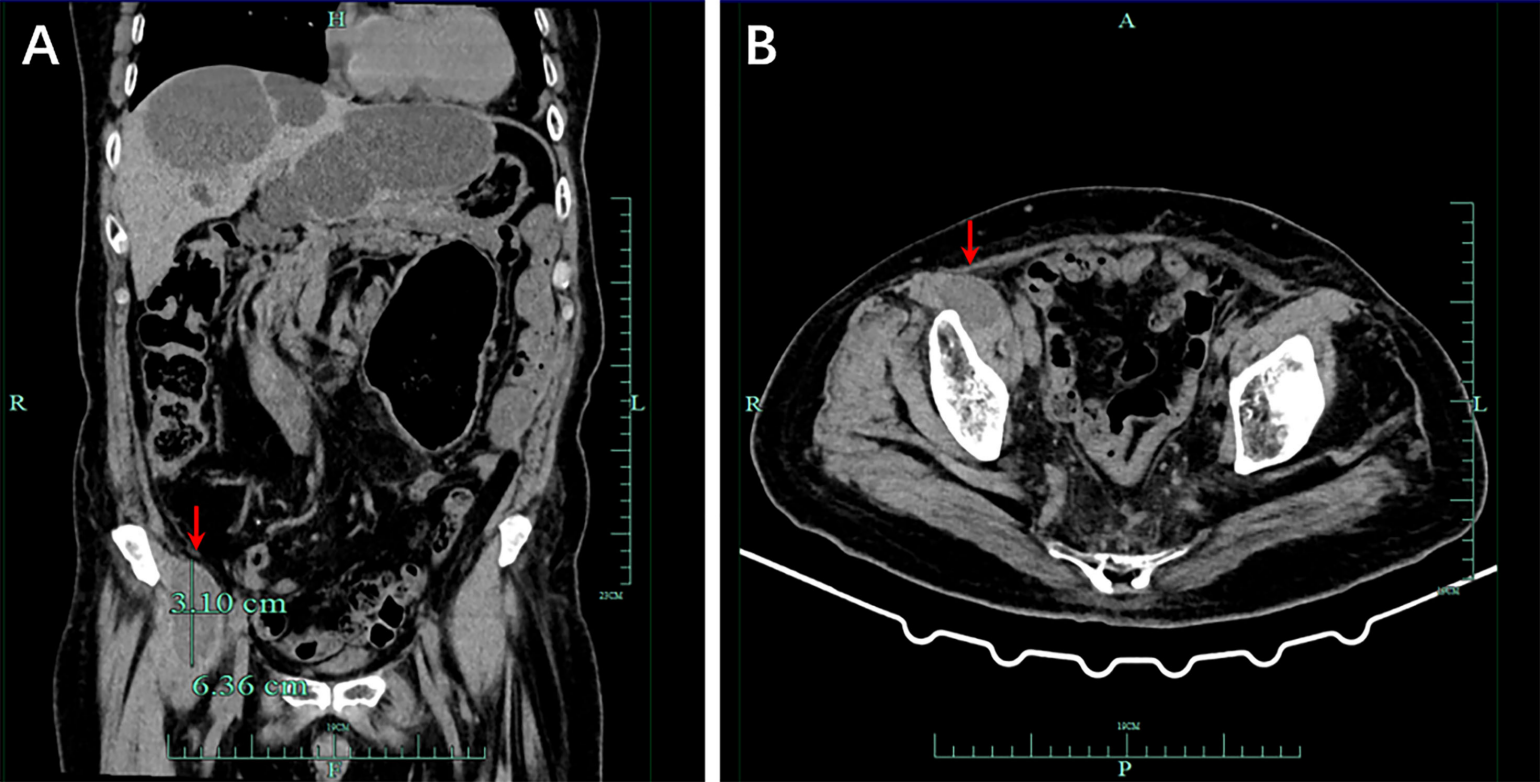
Computed tomography (CT) demonstrated a malignant tumor in the right groin area, and the size of the mass was approximately 6.3 cm × 3.1 cm × 2.0 cm. **(A)** Imaging in the coronal section. **(B)** Tomography scan in the transverse section of the lesion.

**Figure 4:**
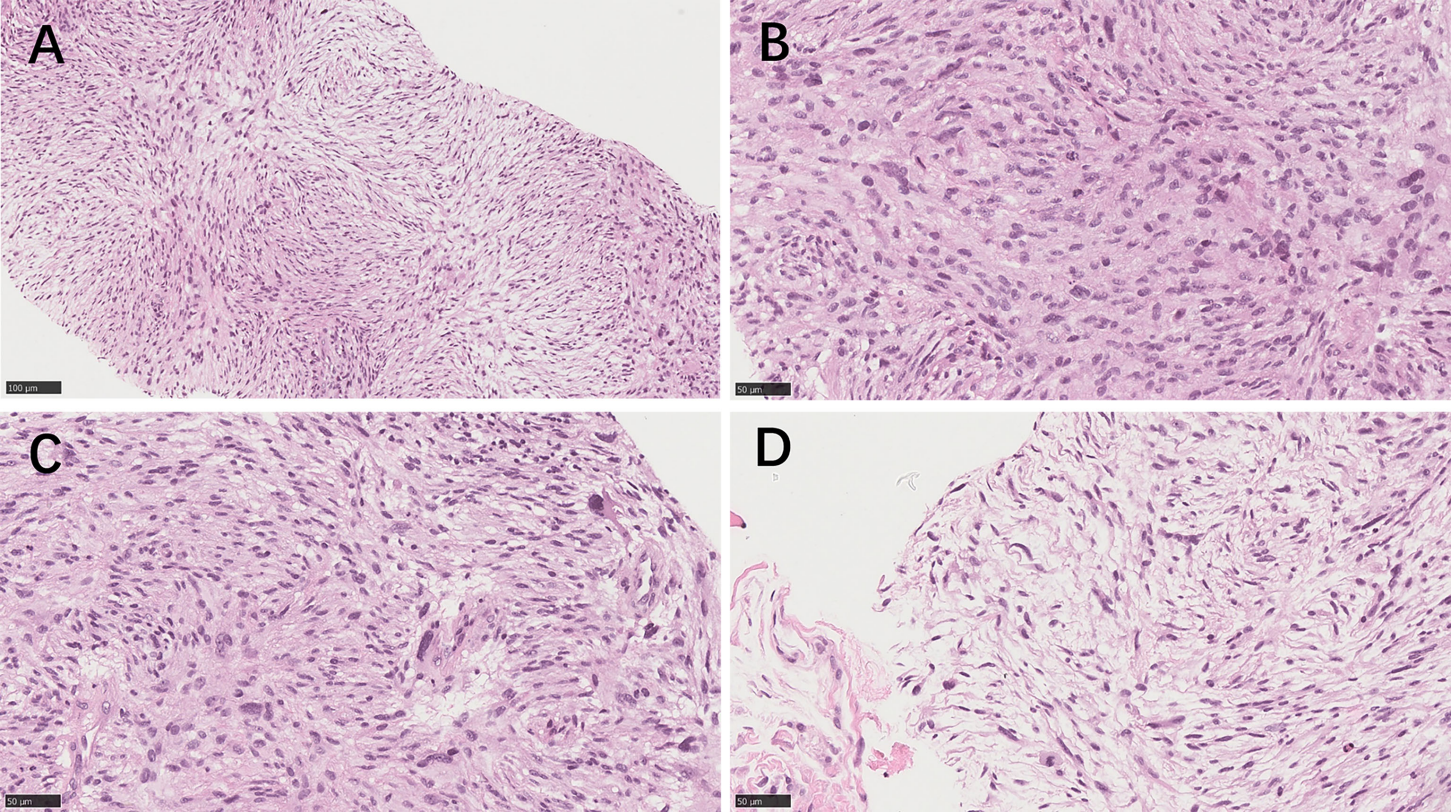
The needle core biopsies of the malignant peripheral nerve sheath tumor. **(A)** Medium‐power image of the tumor area shows spindle-cell tumors with dense arrangement and fascicular hyperplasia (hematoxylin–eosin×200). **(B)** High‐power image of the tumor area shows that the nuclei of the tumor cells are deeply stained, irregular in shape, tadpole-like at the ends, and mitotic figures are readily apparent. **(C)** The tumor cells showed pleomorphism, and large pleomorphic cells were observed in some areas. **(D)** In the sparse cell area, the cell morphology was mostly elongated and wavy. (**B–D**; hematoxylin–eosin×400).

In September 2021, CT showed a new-onset mass in the right groin area of the patient, but the PSA level was within the normal range. We recommend regular CT examination to observe changes in the size of the mass. On November 29, CT (**Figure 3**) revealed that the right inguinal mass was larger than before, and the size of the mass was approximately 6.3 cm × 3.1 cm × 2.0 cm. Needle-core biopsy of the right inguinal mass was performed a week later. Pathological examination revealed spindle-cell tumors (**Figure 4**). The results of immunohistochemistry indicated CD34 (+), CD99 (+), H3K27Me3 (partial deletion), CD117 (-), DOG-1 (-), STAT6 (-), SMA (-), Desmin (-), HMB45 (-), CK5/6 (-), D2-40 (-), MyoD1 (-), S-100 (-), SOX10 (-), PSA (-), Bcl-2 (+), P53 (90% +), and Ki67 (25% +). There were no *SS18* gene rearrangements in the tumor tissues as detected by fluorescence *in situ* hybridization. Additionally, both tumor and blood specimens were collected and prepared for targeted NGS sequencing. The results revealed 41 tumor-specific mutations, including *NF1* and *TP53* mutations, loss of *CDKN2A* expression, and a germline deletion in exon 12 of *MSH2*, with a high tumor mutational burden of 30.9 mutations/MB. Based on these results, malignant peripheral nerve sheath tumor was identified as the patient’s eighth primary malignant tumor. To further define the relationship between malignant peripheral nerve sheath tumors and LS, MMR protein-related immunohistochemistry was performed. Immunohistochemistry of the malignant peripheral nerve sheath tumor was positive for MLH1 and PMS2 but negative for MSH2 and MSH6 (**Figures 2G**–**J**). The results confirmed the presence of LS-associated malignant peripheral nerve sheath tumor. Owing to the old age of the patient and his poor physical ability, surgery was not recommended, and oral medication was indicated. Based on the clinical treatment guidelines, the recommended treatment was intravenous infusion of pembrolizumab combined with oral administration of pazopanib tablets.

## Discussion

The patient was diagnosed with 8 types of multiple primary tumors. After the first tumor was diagnosed, the patient survived for over 41 years and is still alive. To the best of our knowledge, this is the first literature report of malignant peripheral neurilemmoma with LS.

The earliest understanding of multiple primary cancers can be traced back to a report published in 1921. In a survey of 3000 malignant tumors, it was found that approximately 4.7% of patients with tumors have multiple primary cancers (1). Currently, there is no standard reference guide for the diagnostic criteria of multiple primary cancers in clinical practice. The most commonly used diagnostic criteria are provided by the Surveillance Epidemiology and End Results Project (SEER), International Cancer Registration Association, and International Agency for Research on Cancer (IACR/IARC) (5). Epidemiological investigation shows that the incidence of the first primary tumor is increasing year by year due to the aging population, environmental pollution, food safety and other factors. At the same time, the improvement of diagnostic technology, the progress of cancer screening methods and the diversification of anti-tumor treatment methods have affected the incidence of the second primary tumor (1, 6). The exact etiology of most primary tumors is unknown; however, some of the potential risk factors include genetic susceptibility (LS, Li-Fraumeni), immunodeficiency (HIV), chronic infection (hepatitis B), lifestyle (smoking and drinking), and carcinogenic effects of antitumor therapy (1, 2, 7). The diagnosis and differential diagnosis of multiple primary cancers are key conditions to identify the disease. In general, two cancers that are occurring within 6 months are considered simultaneous multiple primary cancers, while those occurring more than 6 months apart are regarded as metachronous multiple primary cancers (1). For the differential diagnosis of multiple primary cancers, pathological, immunohistochemical, and gene detection techniques can be used to identify primary or metastatic secondary cancers. The treatment modality of multiple primary cancers varies according to the case. If there are simultaneous multiple primary cancers, the treatment strategy needs to be discussed at a multidisciplinary meeting to formulate the best treatment plan for the disease at this stage taking into account the other co-existing tumor. However, if it is a metachronous disease and there is only one clinical manifestation of the tumor, an appropriate treatment scheme can be formulated for the existing tumor according to the treatment guidelines of the National Comprehensive Cancer Network. Nevertheless, if the previous primary tumor is still progressing or the clinical symptoms are still obvious, the selection of treatment scheme needs to be discussed at multidisciplinary meetings (8, 9).

Through the analysis of this patient’s disease history, family history and personal history, we considered Lynch syndrome as the risk factor for this patient’s multiple primary cancers. LS, also known as hereditary nonpolyposis colorectal cancer, accounts for approximately 5–8% of all colorectal cancers (10). LS is divided into class I and class II according to the complications by extraintestinal tumors (11). Class I refers to colorectal cancer being the only malignant tumor in the family (12), while class II refers to the occurrence of extraintestinal tumors in addition to colorectal cancer (13). Because of the DNA *MMR* gene mutations, patients with LS are more likely to suffer from all types of cancers than the general population. According to the literature, the extraintestinal tumors are endometrial cancer, ovarian cancer, gastric cancer, cholangiocarcinoma, pancreatic cancer, urothelial carcinoma, bladder cancer, and prostate cancer (14–21). Currently, the screening of LS is a diagnostic standard based on family history and tumor disease history. This mainly follows the Amsterdam and Bethesda criteria, which have different specificities and sensitivities (22). Second, the screening of LS can be also done through the detection of tumor tissues, including the immunohistochemical detection of MMR proteins, polymerase chain reaction, detection of MSI, and *MLH1* methylation analysis. However, the final diagnosis should be confirmed by genetic diagnosis of mutations in germline *MMR*-related genes (including *MLH1*, *MSH2*, *MSH6*, *PMS2*, and *EPCAM* genes) (23). Due to the high cost of genetic testing, misdiagnosis and missed diagnosis rates of LS are extremely high.

Because of the patient’s history of multiple tumors and the drug resistance in prostate cancer after multi-line treatment, NGS examination was suggested to further clarify the underlying causes of multiple tumors and identify new therapeutic targets. Results of the NGS test revealed that the patient had a pathogenic variant of germline deletion in the *MSH2* gene, which confirmed the diagnosis of LS. Previous studies reported that approximately 70–90% of LS cases are due to the deletion of *MLH1* and *MSH2* genes, and 10–30% are due to the deletion of *MSH6* and *PMS2* genes (24, 25). Individuals with *MLH1* or *MSH2* mutations have a high risk of LS type II. Males with *MSH2* mutations have the highest risk of developing various tumors (26). The results of genetic testing showed that the patient in this case report was a high-risk individual with multiple primary tumors. The results of NGS examination showed that a germline deletion in the *MSH2* gene, a high tumor mutational burden of 139.1mutations/MB, and a *TP53* mutation were positive regulatory predictors related to the resistance to the immune checkpoint inhibitor treatment (3). Our alternative treatment plan was administering an immune checkpoint inhibitor combined with anti-vascular-targeted drugs.

In September 2021, during the treatment of prostate cancer, CT examination of the patient revealed a new mass in the right groin. To confirm whether the mass was a metastatic tumor of prostate cancer or a new primary cancer, we performed a needle core biopsy of the right inguinal mass. The tissue was seen as densely arranged, fascicular proliferation of spindle cell tumors under a pathological microscope. Immunohistochemistry results indicated CD34 (+), CD99 (+), and a partial deletion of H3K27Me3. Malignant peripheral nerve sheath tumors are highly malignant, locally aggressive soft tissue sarcomas with nerve sheath differentiation and a high propensity to metastasize. They are rare in the general population, with an approximate lifetime incidence of 0.001% (1/100,000). Up to 50% of all malignant peripheral nerve sheath tumors occur in neurofibromatosis type 1 (NF1) (27). They may occur anywhere in the body, but are most often axial in location and are diagnosed based on the histopathologically demonstrated peripheral nerve sheath differentiation (28). One potential model for malignant peripheral nerve sheath tumors development proposes that the loss of biallelic NF1 in nerve sheath precursor cells results in benign neurofibroma formation. Subsequently, loss of CDKN2A promotes the development of an atypical neurofibroma, followed by additional mutations in *EGFR*, *SUZ12*, and/or *TP53*, causing transformation into malignant peripheral nerve sheath tumors (29). The diagnosis of malignant peripheral nerve sheath tumor is difficult. Molecular detection and gene detection are helpful for a definite diagnosis. The NGS results of the tumor tissue of the reported patient showed a deletion of *CDKN2A* and mutations in the tumor-specific genes *NF1* and *TP53*, which were consistent with the diagnosis of malignant peripheral nerve sheath tumor. During diagnosis, we also confirmed the diagnosis of the tumors as LS-associated extraintestinal malignancies by Immunohistochemical results of MMR protein expression in tissue sections of the prostate and malignant peripheral nerve sheath tumors. The most common tumors in LS are colorectal tumors and endometrial tumors. However, patients with LS and *MMR* gene mutations have a higher risk of developing multiple extraintestinal tumors than the general population. The pathogenesis of multiple extraintestinal tumors can be further clarified by detecting MMR protein expression in tumor tissues. Owing to the long history of the case and the lack of preservation of pathological tissues in other hospitals, we could not perform *MMR* gene-related tests for the nasal tumors and skin cancer.

Studies have reported that patients with LS have a very low probability of developing colon cancer before the age of 25 (30, 31). However, progression from colonic adenomas to cancer is more rapid in patients with LS than in the general population. Therefore, surveillance colonoscopy is recommended every 1-2 years in patients with a family history of colon cancer (32). The patient reported in this case report was highly concerned about his health. Since his parents had a history of colon cancer, he actively requested gastrointestinal endoscopy during his first visit when he was complaining of abdominal discomfort. After the first diagnosis of colon cancer, the patient underwent regular gastroenterological endoscopy examinations every year. Gastroenterological endoscopy is the only monitoring program that has proven effective in reducing the mortality of LS-related colorectal cancer (33). When a germline mutation of the *MSH2* gene was found in the first NGS gene examination and the diagnosis of LS was confirmed, it was strongly recommended that the relatives of the patient undergo gene examination and regular gastroenterological endoscopy (34, 35). Unfortunately, clinicians pay less attention to extraintestinal tumors of LS. The patient had an advanced prostate cancer at the time of diagnosis and had poor treatment outcomes. Therefore, we suggest that patients with LS undergo a general physical examination in addition to colonoscopy monitoring and visit the doctor whenever they feel uncomfortable. Upon regular systemic examination and monitoring of this patient, we detected a new lesion early and it was promptly diagnosed. Currently, we do not consider a strong treatment protocol for this patient, and consider the will of the patient and his family to choose a treatment that is based on prolonging the patient’s survival and improving his quality of life.

## Conclusion

In conclusion, this is the first report of a malignant peripheral nerve sheath tumor in LS. Through follow-up and investigation of this case, we consider that the main risk factor for the multiple primary tumors in this patient was LS. More attention should be given by clinicians to LS-associated extracolonic tumors. At the same time, this case highlights the importance of clinical periodic general physical examination in patients with multiple primary tumors with LS. For patients with LS, we recommend that multiple primary tumors undergo *MMR* gene-related testing to identify the underlying cause of the tumor. Because of the high incidence of tumors in patients with LS, regular general physical checkups, early detection, early diagnosis, and modest treatment instead of overtreatment are important for patients with LS to improve their survival rate.

## Data Availability Statement

The datasets presented in this article are not publicly available because of privacy or ethical restrictions. Requests to access the datasets should be directed to the corresponding author upon reasonable request.

## Ethics Statement

Written informed consent was obtained from the individual(s) for the publication of any potentially identifiable images or data included in this article.

## Author Contributions

JJ, TH, XL, XY, LH, ZY and LT collected the clinical information, diagnostic information, therapeutic information, and images of the patients. XR, JL, MK and LL described the pathological diagnosis. JJ, XL and YZ wrote the article. JJ, MX and YC translated the article. JHL, YZZ and ZL revised the article. SL identified the case and proofread the article. JJ, TH and XL have contributed equally to this work. All authors contributed to the article and approved the submitted version.

## Funding

This work was supported by the construction fund of medical key disciplines of Hangzhou (2020SJZDXK004), the program of Zhejiang Provincial TCM Sci-tech Plan (2020ZQ041), and Zhejiang SL famous traditional Chinese medicine expert inheritance studio project (GZS202002).

## Conflict of Interest

The authors declare that the research was conducted in the absence of any commercial or financial relationships that could be construed as a potential conflict of interest.

## Publisher’s Note

All claims expressed in this article are solely those of the authors and do not necessarily represent those of their affiliated organizations, or those of the publisher, the editors and the reviewers. Any product that may be evaluated in this article, or claim that may be made by its manufacturer, is not guaranteed or endorsed by the publisher.

## References

[1] Vogt A Schmid S Heinimann K Frick H Herrmann C Cerny T. Multiple Primary Tumours: Challenges and Approaches, a Review. ESMO Open (2017) 2(2):e000172. doi: 10.1136/esmoopen-2017-000172 28761745 PMC5519797

[2] Copur MS Manapuram S. Multiple Primary Tumors Over a Lifetime. Oncology (2019) 33(7):629384. 31365752

[3] Markakis C Marinis A Dikeakos P Grivas P Voultsos M Liarmakopoulos E. Multiple Synchronous Primary Neoplasms of the Breast, Colon and Rectum After Surgery for Endometrial Cancer: A Case Report. Int J Surg Case Rep (2013) 4(5):493–5. doi: 10.1016/j.ijscr.2013.01.001 PMC3731706 23562900

[4] Duraturo F Liccardo R De Rosa M Izzo P. Genetics, Diagnosis and Treatment of Lynch Syndrome: Old Lessons and Current Challenges. Oncol Lett (2019) 17(3):3048–54. doi: 10.3892/ol.2019.9945 PMC6396136 30867733

[5] Coyte A Morrison DS McLoone P. Second Primary Cancer Risk - the Impact of Applying Different Definitions of Multiple Primaries: Results From a Retrospective Population-Based Cancer Registry Study. BMC Cancer (2014) 14:272. doi: 10.1186/1471-2407-14-272 24742063 PMC4005906

[6] Herrmann C Cerny T Savidan A Vounatsou P Konzelmann I Bouchardy C. Cancer Survivors in Switzerland: A Rapidly Growing Population to Care for. BMC Cancer (2013) 13:287. doi: 10.1186/1471-2407-13-287 23764068 PMC3685597

[7] De Luca A Frusone F Vergine M Cocchiara R La Torre G Ballesio L. Breast Cancer and Multiple Primary Malignant Tumors: Case Report and Review of the Literature. In Vivo (2019) 33(4):1313–24. doi: 10.21873/invivo.11605 PMC6689364 31280224

[8] Zhang Z Gao S Mao Y Mu J Xue Q Feng X. Surgical Outcomes of Synchronous Multiple Primary Non-Small Cell Lung Cancers. Sci Rep (2016) 6:23252. doi: 10.1038/srep23252 27254665 PMC4890551

[9] Heroiu Cataloiu AD Danciu CE Popescu CR. Multiple Cancers of the Head and Neck. Maedica (2013) 8:80–5. PMC3749768 24023604

[10] Hampel H Frankel WL Martin E Arnold M Khanduja K Kuebler P. Feasibility of Screening for Lynch Syndrome Among Patients With Colorectal Cancer. J Clin Oncol (2008) 26(35):5783–8. doi: 10.1200/JCO.2008.17.5950 PMC2645108 18809606

[11] Sinicrope FA. Lynch Syndrome-Associated Colorectal Cancer. N Engl J Med (2018) 379(8):764–73. doi: 10.1056/NEJMcp1714533 30134129

[12] Vasen HF Mecklin JP Khan PM Lynch HT. The International Collaborative Group on Hereditary Non-Polyposis Colorectal Cancer (ICG-HNPCC). Dis Colon Rectum (1991) 34(5):424–5. doi: 10.1007/BF02053699 2022152

[13] Park JG Vasen HF Park KJ Peltomaki P Ponz de Leon M Rodriguez-Bigas MA. Suspected Hereditary Nonpolyposis Colorectal Cancer: International Collaborative Group on Hereditary Non-Polyposis Colorectal Cancer (ICG-HNPCC) Criteria and Results of Genetic Diagnosis. Dis Colon Rectum (1999) 42(6):710–5; discussion 715-6. doi: 10.1007/BF02236922 10378593

[14] Yokoyama T Takehara K Sugimoto N Kaneko K Fujimoto E Okazawa-Sakai M. Lynch Syndrome-Associated Endometrial Carcinoma With MLH1 Germline Mutation and MLH1 Promoter Hypermethylation: A Case Report and Literature Review. BMC Cancer (2018) 18(1):576. doi: 10.1186/s12885-018-4489-0 29783979 PMC5963021

[15] Ring KL. Ovarian Cancer Risk in Lynch Syndrome: It's Time to Individualise. BJOG (2021) 128(4):737. doi: 10.1111/1471-0528.16481 32892474

[16] Skeldon Sean C Semotiuk K Aronson M Holter S Gallinger S Pollett A. Patients With Lynch Syndrome Mismatch Repair Gene Mutations are at Higher Risk for Not Only Upper Tract Urothelial Cancer But Also Bladder Cancer. Eur Urol (2013) 63(2):379–85. doi: 10.1016/j.eururo.2012.07.047 22883484

[17] Hendifar AE Larson BK Rojansky R Guan M Gong J Placencio V. Pancreatic Cancer 'Mismatch' in Lynch Syndrome. BMJ Open Gastroenterol (2019) 6(1):e000274. doi: 10.1136/bmjgast-2019-000274 PMC6577306 31275582

[18] Haraldsdottir S Hampel H Wei L Wu C Frankel W Bekaii-Saab T. Prostate Cancer Incidence in Males With Lynch Syndrome. Genet Med (2014) 16(7):553–7. doi: 10.1038/gim.2013.193 PMC4289599 24434690

[19] Grobet-Jeandin E Pinar U Rouprêt M. Upper Urinary Tract Urothelial Carcinoma in Lynch Syndrome Patients: The Urologist Still Has a Role in Genetic Screening. Eur Urol Oncol (2022) 5(1):42–3. doi: 10.1016/j.euo.2021.12.004 34980573

[20] Boland CR Yurgelun MB Mraz KA Boland PM. Managing Gastric Cancer Risk in Lynch Syndrome: Controversies and Recommendations. Fam Cancer (2022) 21(1):75–8. doi: 10.1007/s10689-021-00235-3 PMC8799584 33611683

[21] Ando Y Kumamoto K Matsukawa H Ishikawa R Suto H Oshima M. Low Prevalence of Biliary Tract Cancer With Defective Mismatch Repair Genes in a Japanese Hospital-Based Population. Oncol Lett (2022) 23(1):4. doi: 10.3892/ol.2021.13122 34820003 PMC8607234

[22] Singh S Resnick KE. Lynch Syndrome and Endometrial Cancer. South Med J (2017) 110(4):265–9. doi: 10.14423/SMJ.0000000000000633 28376523

[23] Cox VL Saeed Bamashmos AA Foo WC Gupta S Yedururi S Garg N. Lynch Syndrome: Genomics Update and Imaging Review. Radiographics (2018) 38(2):483–99. doi: 10.1148/rg.2018170075 29528821

[24] Weissman SM Burt R Church J Erdman S Hampel H Holter S. Identification of Individuals at Risk for Lynch Syndrome Using Targeted Evaluations and Genetic Testing: National Society of Genetic Counselors and the Collaborative Group of the Americas on Inherited Colorectal Cancer Joint Practice Guideline. J Genet Couns (2012) 21(4):484–93. doi: 10.1007/s10897-011-9465-7 22167527

[25] Egoavil C Alenda C Castillejo A Paya A Peiro G Sánchez-Heras AB. Prevalence of Lynch Syndrome Among Patients With Newly Diagnosed Endometrial Cancers. PloS One (2013) 8(11):e79737. doi: 10.1371/journal.pone.0079737 24244552 PMC3820559

[26] Ramsoekh D Wagner A van Leerdam ME Dooijes D Tops CM Steyerberg EW. Cancer Risk in MLH1, MSH2 and MSH6 Mutation Carriers; Different Risk Profiles may Influence Clinical Management. Hered Cancer Clin Pract (2009) 7(1):17. doi: 10.1186/1897-4287-7-17 20028567 PMC2804564

[27] Amirian ES Goodman JC New P Scheurer ME. Pediatric and Adult Malignant Peripheral Nerve Sheath Tumors: An Analysis of Data From the Surveillance, Epidemiology, and End Results Program. J Neurooncol (2014) 116(3):609–16. doi: 10.1007/s11060-013-1345-6 24390465

[28] Ferrari A Bisogno G Carli M. Management of Childhood Malignant Peripheral Nerve Sheath Tumor. Paediatr Drugs (2007) 9(4):239–48. doi: 10.2165/00148581-200709040-00005 17705563

[29] Prudner BC Ball T Rathore R Hirbe AC. Diagnosis and Management of Malignant Peripheral Nerve Sheath Tumors: Current Practice and Future Perspectives. Neurooncol Adv (2019) 2(Suppl 1):i40–9. doi: 10.1093/noajnl/vdz047 PMC7317062 32642731

[30] Quehenberger F Vasen HF van Houwelingen HC. Risk of Colorectal and Endometrial Cancer for Carriers of Mutations of the Hmlh1 and Hmsh2 Gene: Correction for Ascertainment. J Med Genet (2005) 42(6):491–6. doi: 10.1136/jmg.2004.024299 PMC1736072 15937084

[31] Hampel H Stephens JA Pukkala E Sankila R Aaltonen LA Mecklin JP. Cancer Risk in Hereditary Nonpolyposis Colorectal Cancer Syndrome: Later Age of Onset. Gastroenterology (2005) 129(2):415–21. doi: 10.1016/j.gastro.2005.05.011 16083698

[32] de Jong AE Hendriks YM Kleibe uker JH de Boer SY Cats A Griffioen G. Decrease in Mortality in Lynch Syndrome Families Because of Surveillance. Gastroenterology (2006) 130(3):665–71. doi: 10.1053/j.gastro.2005.11.032 16530507

[33] Mecklin JP Aarnio M Läärä E Kairaluoma MV Pylvänäinen K Peltomäki P. Development of Colorectal Tumors in Colonoscopic Surveillance in Lynch Syndrome. Gastroenterology (2007) 133(4):1093–8. doi: 10.1053/j.gastro.2007.08.019 17919485

[34] Vasen HF Möslein G Alonso A Bernstein I Bertario L Blanco I. Guidelines for the Clinical Management of Lynch Syndrome (Hereditary non-Polyposis Cancer). J Med Genet (2007) 44(6):353–62. doi: 10.1136/jmg.2007.048991 PMC2740877 17327285

[35] Vasen HF Blanco I Aktan-Collan K Gopie JP Alonso A Aretz S. Revised Guidelines for the Clinical Management of Lynch Syndrome (HNPCC): Recommendations by a Group of European Experts. Gut (2013) 62(6):812–23. doi: 10.1136/gutjnl-2012-304356 PMC3647358 23408351

